# A cell engineering approach to enzyme-based fed-batch fermentation

**DOI:** 10.1186/s12934-021-01634-y

**Published:** 2021-07-24

**Authors:** Michael Sibley, John M. Ward

**Affiliations:** Department of Biochemical Engineering, UCL, Gower Street, London, WC1E 6BT UK

**Keywords:** Enzyme-based fed-batch fermentation, Bacterial glucoamylase, Starch to glucose conversion, Cell engineering for bioprocess

## Abstract

**Background:**

A fundamental problem associated with *E. coli* fermentations is the difficulty in achieving high cell densities in batch cultures, attributed in large part to the production and accumulation of acetate through a phenomenon known as overflow metabolism when supplying enough glucose for the cell density desired. Although a fed-batch configuration is the standard method for reducing such issues, traditional fed-batch systems require components which become problematic when applying them at smaller scale. One alternative has been the development of a system whereby the enzymatic degradation of starch is used to release glucose at a controlled rate. However, to date, amylolytic enzymes have only been applied to the culture exogenously, whereas our goal is to design and construct a self-secreting amylolytic chassis capable of self-regulated enzyme-based fed-batch fermentation.

**Results:**

A putative glucoamylase from *C. violaceum* has been cloned and expressed in *E. coli* BL21(DE3) and W3110, which exhibits significant glucose releasing amylolytic activity. Extracellular amylolytic activity was enhanced following a replacement of the enzymes native signal peptide with the DsbA signal sequence, contributing to a glucoamylase secreting strain capable of utilising starch as a sole carbon source in defined media. Introduction of *PcstA*, a glucose sensitive K12 compatible promoter, and the incorporation of this alongside *C. violaceum* glucoamylase in *E. coli* W3110, gave rise to increased cell densities in cultures grown on starch (OD_600_ ∼ 30) compared to those grown on an equivalent amount of glucose (OD_600_ ∼ 15). Lastly, a novel self-secreting enzyme-based fed-batch fermentation system was demonstrated via the simultaneous expression of the *C. violaceum* glucoamylase and a recombinant protein of interest (eGFP), resulting in a fourfold increase in yield when grown in media containing starch compared with the glucose equivalent.

**Conclusions:**

This study has developed, through the secretion of a previously uncharacterised bacterial glucoamylase, a novel amylolytic *E. coli* strain capable of direct starch to glucose conversion. The ability of this strain to achieve increased cell densities as well as an associated increase in recombinant protein yield when grown on starch compared with an equivalent amount of glucose, demonstrates for the first time a cell engineering approach to enzyme-based fed-batch fermentation.

**Supplementary Information:**

The online version contains supplementary material available at 10.1186/s12934-021-01634-y.

## Background

Overflow metabolism is a major contributory factor limiting cell densities and recombinant protein yields in *E. coli* fermentations. During rapid glucose uptake in aerobic conditions, the flux of acetyl-coA through *E. coli* central metabolism is diverted away from the tricarboxylic acid (TCA) cycle and towards the production of acetate [1]. The resulting accumulation of acetate lowers media pH, leading to both a reduction in growth [2] and a reduction in recombinant protein yield [3]. To achieve the cell densities required for many purposes batch growth, where all the glucose is added to the media at the start, will lead to overflow metabolism and acetate build up. By limiting the availability of glucose, the specific growth rate of cells within the culture can be reduced below the threshold required to initiate overflow metabolism, therefore minimising the production and accumulation of acetate. This concept of substrate limited fed-batch fermentation is a commonly used method to minimise the detrimental effects of overflow metabolism and increase cell densities and recombinant protein yields. Traditional systems vary extensively in the manner of substrate delivery; some are programmed with pre-determined feed rates e.g. constant or exponential, while others rely on automatic feedback control [4]. Regardless of the mechanism, traditional fed-batch fermentation is often an impractical solution for smaller scale fermentations such as those in shake flasks, owing to the sophistication of the control systems required, as well as other technical difficulties including unsatisfactory flow and mixing of the small, concentrated feed volumes. Many of these issues are being challenged through the development and increased availability of miniature bioreactors, for example the ambr® 250 from Sartorius or the DASbox® from Eppendorf, however shake flasks still provide an inexpensive and effective way of reproducibly performing many types of industrially-relevant cell cultivations for process development, and are therefore still widely used across industry and academia [5].

An alternative approach to the traditional fed-batch fermentation concept, and one which can be applied directly to shake flasks, has been the development of substrate delivery via an internal supply mechanism [6, 7]. An example specific to glucose supply uses silicone elastomer discs containing glucose crystals which, when immersed in media, release glucose by diffusion [8]. Another method has been the development of enzyme-based fed-batch fermentation, in which glucose is provided to the culture via the enzymatic degradation of starch i.e. metabolically inactive polysaccharide is converted into metabolically active monosaccharide following the addition of an amylolytic enzyme [9, 10]. This technology, referred to as EnBase®, has been commercially developed by BioSilta [11] as EnPresso® media, and uses an amyloglucosidase or glucoamylase (E.C.3.2.1.3) isolated from *Aspergillus niger* to convert starch to glucose, with the rate of glucose release determined by the concentration of glucoamylase supplied to the culture. Original experimental work used a two phase media system consisting of mineral salt medium (MSM) combined with starch infused agar layers [10], providing sufficient concentrations of starch to achieve high cell densities while also avoiding issues associated with solubility. The starch infused agar layers allowed starch to continuously dissolve into the media over time. Developers were able to infuse gels with 100 g/L starch, which, based on the assumption that one gram of glucose could yield approximately 0.5 g dry cell weight, they calculated would be enough to give a theoretical cell density of 30 g/L dry cell weight, equivalent to an OD_600_ of approximately 100. Optimised glucoamylase concentrations gave rise to cell densities with OD_600_ values of 20 to 30, or approximately 6 to 9 g/L dry cell weight, a significant advantage over more traditionally used complex media such as LB [10].

Despite these advantages, systems to date have been limited as they require the addition of an externally supplied amylolytic enzyme. In this study we describe the development and application of a novel self-secreting amylolytic *E. coli* strain, providing the first demonstrated example of a cell engineering approach to enzyme-based fed-batch fermentation. The scope of the work described here includes the identification and characterisation of a previously unreported bacterial glucoamylase cloned from *Chromobacterium violaceum*, the regulated expression and secretion of this glucoamylase from *E. coli*, and finally the application of this amylolytic strain to enhance shake flask bioprocesses resulting in increased cell densities and recombinant protein yield.

## Results

### Identification and characterisation of novel bacterial glucoamylases

**Fig. 1:**
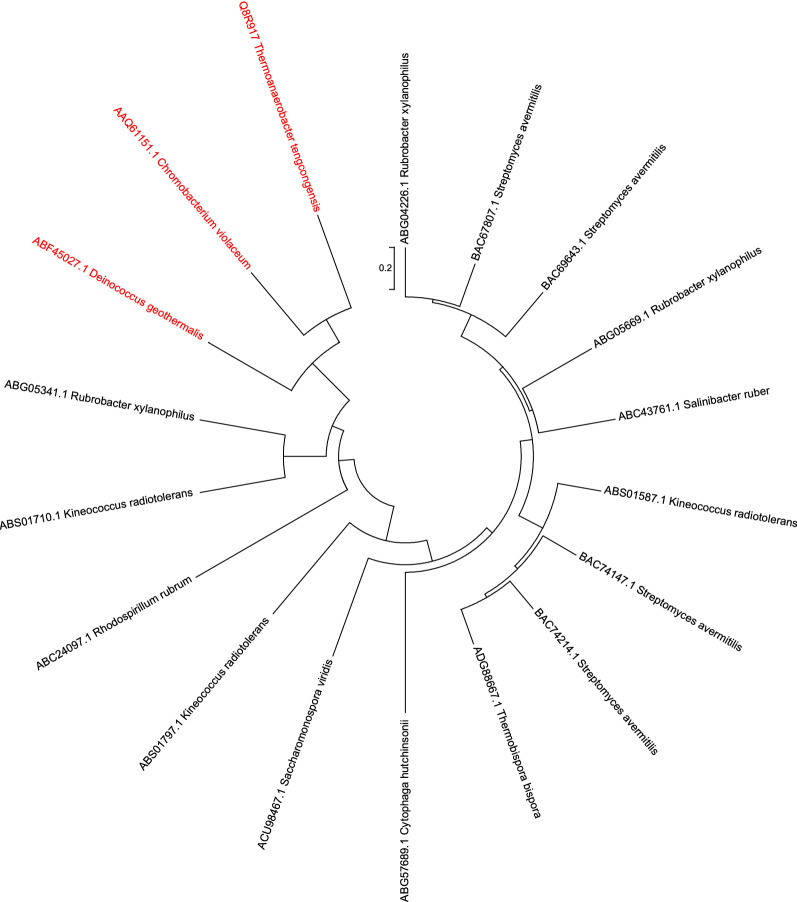
Phylogenetic tree of putative bacterial glucoamylases. Putative bacterial glucoamylases cross referenced with organisms available in-house. Enzymes selected for initial screening (red) due to their relatedness to the previously characterised *T. tengcongensis* glucoamylase. Phylogenetic tree created using neighbour-joining method by Molecular Evolutionary Genetics Analysis version 7.0 (MEGA7) software

**Fig. 2:**
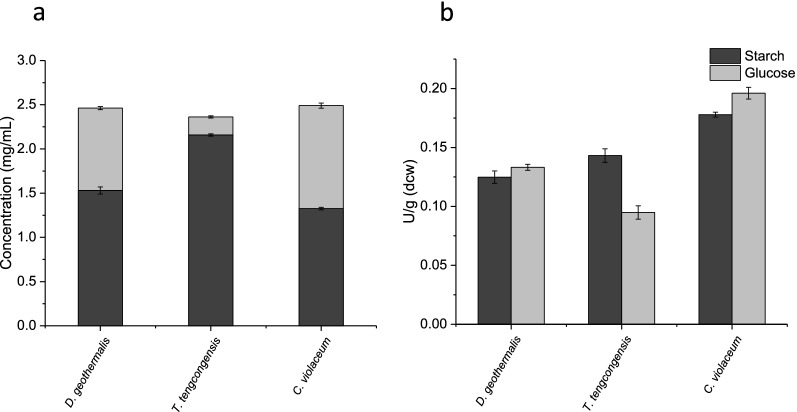
Starch degradation and glucose accumulation from cell free extracts of *D. geothermalis, T. tengcongensis* and *C. violaceum* glucoamylases. Cell free extracts from BL21(DE3) harbouring either pQR1706 (*D. geothermalis* glucoamylase, pQR1707 (*T. tengcongensis* glucoamylase), or pQR1708 (*C. violaceum* glucoamylase), induced with 0.4 mM IPTG at an OD_600_ of 0.8 and grown in TB for 24 h at 25 °C, 180 rpm. **a** Concentration (mg/mL) of remaining starch (dark grey) and accumulated glucose (light grey) following 1 h of amylase assay for each CFE. **b** Starch degrading activity in units (dark grey) defined as the disappearance of 1 mg/mL of starch/iodine complex per minute per gram of dry cell weight, and glucose accumulating activity in units (light grey) defined as accumulation of 1 mg/mL of glucose per minute per gram of dry cell weight, for each CFE. Starch degradation in amylase assay determined by the change in absorbance of starch/iodine complex at 600 nm, glucose accumulation measured using HPAEC-PAD analysis. Error bars represent standard deviation, n = 3

Glucoamylases are able to successively hydrolyse the terminal α-1,4 glycosidic links from the non-reducing ends of polysaccharides [12], predominantly generating glucose as opposed to other maltodextrins. They are the enzyme of choice for current enzyme-based fed-batch fermentation systems, however, the majority of previously characterised glucoamylases, and those which are primarily used for industrial purposes, are of fungal origin, largely from *Aspergillus niger* and *Rhizopus oryzae* [13]. For the most part, fungal glucoamylases are highly glycosylated, which is thought to be important for their stability [14], meaning they are often difficult to successfully express in *E. coli*. A notable exception being the heavily glycosylated glucoamylase STA1 from *Saccharomyces cerevisiae* (var. *diastaticus*), which has been successfully expressed in *E. coli* previously as a non-glycosylated version [15]. However, owing to the lack of examples in the literature, it seemed appropriate to identify putative bacterial glucoamylases, to facilitate expression and secretion within an *E. coli* host. One of the few examples of a bacterially derived glucoamylase which has previously been expressed in *E. coli* is the thermostable glucoamylase (TtcGA) from *Thermoanaerobacter tengcongensis* MB4 [16]. Using the carbohydrate-active enzymes (CAZy) database [17], putative bacterial glucoamylases from Glycoside Hydrolase Family 15 were identified and selected based on their relatedness to the *T. tengcongensis* glucoamylase [Molecular Evolutionary Genetics Analysis version 7.0 (MEGA7) software] the relationship of these bacterial glucoamylases is shown using the tree in Fig. 1. Two novel glucoamylases from *D. geothermalis* and *C. violaecum* were then cloned along with the *T. tengcongensis* derived glucoamylase, into pET29a and pET28a expression vectors. We found no differences in expression when either pET28 or pET29 was used. The relationship between the three glucoamylases studied in detail here (the ones in red in Fig. 2) are shown in the alignment in Additional file 1: Fig. S1.

**Fig. 3:**
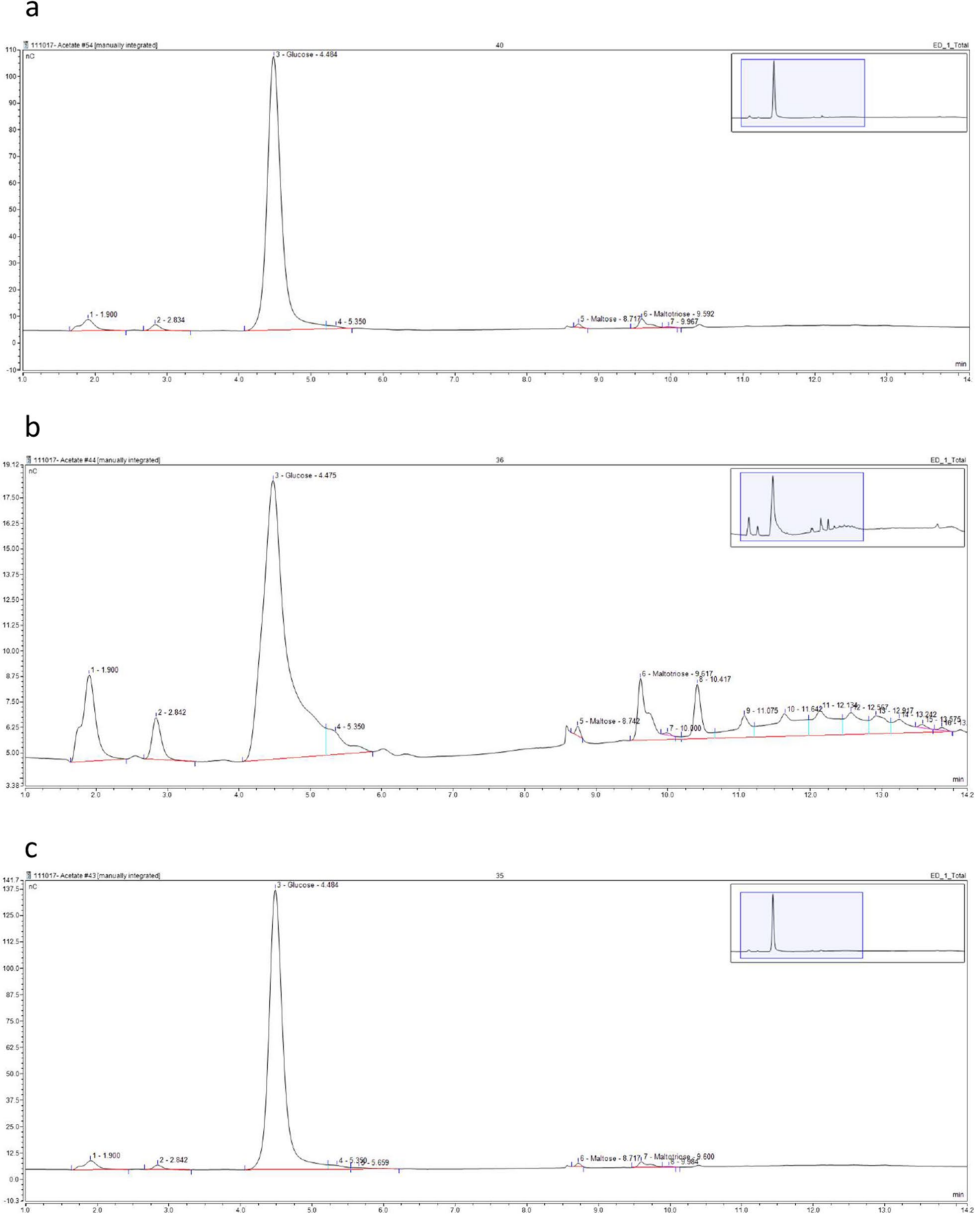
Chromatograms of *D. geothermalis, T. tengcongensis* and *C. violaceum* glucoamylase hydrolysis products. Example ICS chromatograms for amylase assay samples removed after 1 h, for clarified lysates of *E. coli* BL21(DE3) expressing **a** pQR1706, **b** pQR1707, and **c** pQR1708, cultured in TB for 24 h at 25 °C, 180 rpm (induced with 0.4 mM IPTG at an OD_600_ of 0.8)

Both starch degradation and glucose accumulation assays were used to determine cell free extract activities for *D. geothermalis*, *T. tengcongensis*, and *C. violaecium* glucoamylases. Each enzyme produced glucose as the predominant hydrolysis product, with the combined mass of remaining starch and accumulated glucose at an assay time of 1 h approximately equalling the initial concentration of starch (2.5 mg/mL) within the assay (Fig. 2a). A disparity between the rates of starch degradation and glucose accumulation was evident for the *T. tengcongesis* glucoamylase, but not for the *D. geothermalis* or the *C. violaecium* glucoamylases (Fig. 2b). The increased rate of starch degradation compared with glucose accumulation seen with the *T. tengcongensis* glucoamylase indicated that the latter two enzymes had lower endo-acting activity, and a cleaner hydrolysis product profile (Fig. 3).

**Fig. 4:**
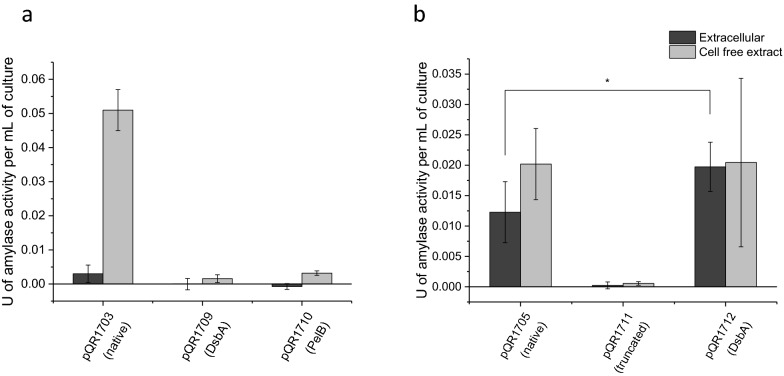
Extracellular and cell free extract activities of *D. geothermalis* and *C. violaceum* glucoamylases following addition of exogenous signal sequences. Extracellular (dark grey) and cell free extract (light grey) activities expressed in units (defined as the disappearance of 1 mg/mL of starch/iodine complex per minute) per mL of culture. Constructs expressed in BL21(DE3) and cultured for 24 h at 25 °C, 180 rpm in LB (induced with 0.4 mM IPTG at an OD_600_ of 0.6–0.8). **a** pQR1703 (*D. geothermalis* glucoamylase), pQR1709 (*D. geothermalis* glucoamylase containing DsbAss) or pQR1710 (*D. geothermalis* glucoamylase containing PelB signal sequence). **b** pQR1705 (*C. violaceum* glucoamylase), pQR1711 (truncated *C. violaceum* glucoamylase) or pQR1712 (*C. violaceum* glucoamylase containing DsbA signal sequence). Error bars represent standard deviation, n = 6

With both the *D. geothermalis* and *C. violaecium* glucoamylases giving rise to suitable hydrolysis product profiles, each of the enzymes were investigated for their secretory potential based on targeting them to the periplasm and relying on outer membrane permeability for release into the extracellular media. If present, native signal sequences were examined, as was the addition of exogenous signal peptides DsbA (originally derived from thiol disulfide oxidoreductase found in many bacterial species including *E. coli*) [18, 19] or PelB (originally derived from the pectate lyase B from *Pectobacterium carotovorum*) [20], directing proteins through the SRP [21] or Sec [22] pathways respectively. The *D. geothermalis* glucoamylase did not contain a native secretory signal sequence (SignalP 4.1) and as a result did not display any extracellular activity (Fig. 4a). Neither the addition of exogenous signal sequences DsbA (pQR1709) or PelB (pQR1710), increased extracellular activity. In fact, their additions diminished cell free extract activities. In contrast, the *C. violaecium* glucoamylase did contain a native secretory signal sequence and displayed extracellular activity without modification (Fig. 4b). This activity was eliminated following the removal of the native signal sequence (pQR1711), but rescued with a marginal improvement over the original following the addition of DsbA signal sequence (pQR1712).

**Fig. 5:**
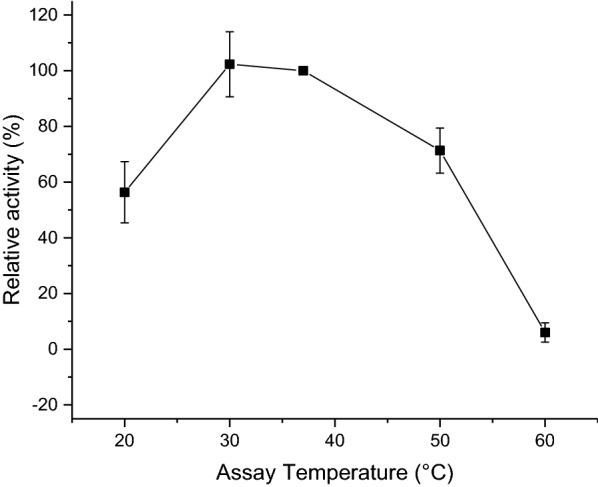
Relative activity of *C. violaceum* glucoamylase with temperature. Extracellular activity of *C. violaceum* glucoamylase at varying temperatures (20 °C to 60 °C) expressed as a percentage of the activity from the standard amylolytic assay carried out at 37 °C. Extracellular media samples for assays extracted from BL21(DE3) harbouring pQR1705 (*C. violaceum* glucoamylase) cultured in LB for 24 h at 25 °C, 180 rpm (induced with 0.4 mM IPTG at an OD_600_ of 0.6–0.8). Activities within standard amylase assay determined by the change in absorbance of starch/iodine complex at 600 nm. Error bars represent standard deviation, n = 3

To ensure extracellularly located *C. violaceum* glucoamylase remained active at typical *E. coli* culture temperatures, starch degradation was measured using amylase assays at temperatures ranging from 20 to 60 °C (Fig. 5). The enzyme’s optimal activity was found to be at approximately 30 °C, while still retaining 50% activity at 20 °C.

### Utilisation of starch as a sole carbon source by a glucose releasing amylolytic *E. coli* strain

**Fig. 6:**
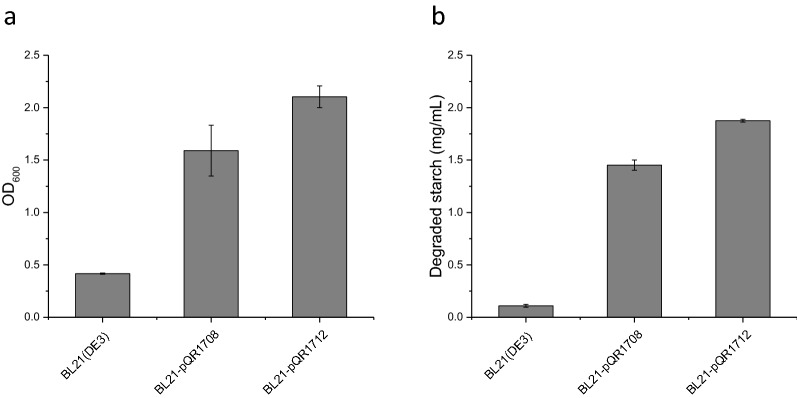
Glucose releasing amyloltic *E. coli* strains and their utilisation of starch as a sole carbon source. *E. coli* BL21(DE3) harbouring either pQR1708 (native signal sequence) or pQR1712 (DsbA signal sequence) inoculated with an initial OD_600_ of 0.25 and cultured over a 24 h period at 30 °C, 250 rpm, in glucose free MSM supplemented with 2.5 mg/mL starch. **a** Cell density (OD_600_) after 24 h of culture. **b** Amount of starch degraded (mg/mL) in culture media after 24 h of culture, determined by absorbance of starch/iodine complex at 600 nm in starch degradation assay. Error bars represent standard deviation, n = 3

*E. coli* BL21(DE3) expressing the *C. violaceum* glucoamylase was able to grow in glucose free MSM containing starch as a sole carbon source (Fig. 6a). A culture period of 24 h led to OD_600_ values in excess of 1.5 for both pQR1708 (native signal sequence) and pQR1712 (DsbA signal sequence) grown in glucose free MSM containing 2.5 mg/mL starch. Conversely the BL21(DE3) control cultures did not achieve densities far beyond that of the initial inoculum density (OD_600_ of 0.25). Alongside increases in cell density, starch degradation within the glucose free MSM was also significantly greater in the two glucoamylase expressing strains compared with the untransformed control, with over half of the available starch (> 1.5 mg/mL) degraded in 24 h (Fig. 6b). A similar growth advantage for plasmid containing cells was seen in cultures of *E. coli* W3110 with and without the a-amylase expressing plasmid pQR187, Additional file 1: Fig S3.

**Fig. 7:**
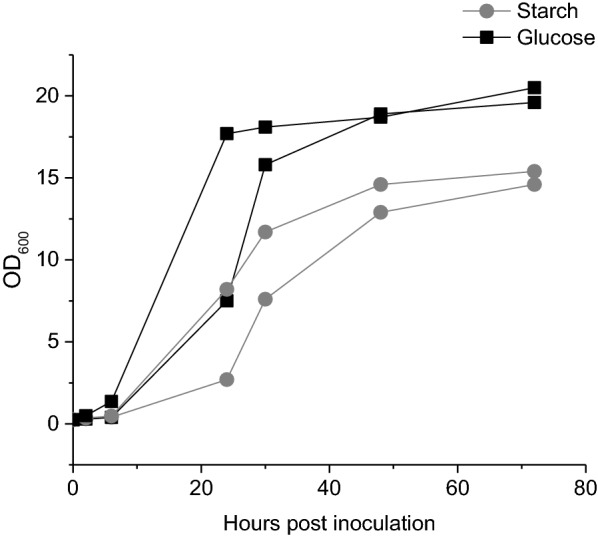
Growth curves for glucoamylase secreting *E. coli* cultured in MSM containing starch or glucose. Growth curves over 72 h for *E. coli* BL21(DE3) harbouring pQR1712 cultured in MSM containing starch–agar layers (circle) or MSM supplemented with 50 mg/mL glucose (square) in duplicate (induced with 0.4 mM IPTG at inoculation [inoculum equivalent to an initial OD_600_ of 0.1)]

Growth characteristics of BL21(DE3) harbouring pQR1712 were further explored using starch/agar layers in shake flasks (Additional file 1: Fig S4), significantly increasing the availability of starch within the glucose free MSM. These cultures achieved cell densities with an OD_600_ of approximately 15 (Fig. 7), equivalent to commonly used complex media such as LB or TB, albeit over a longer culture period of 72 h. However, compared to MSM flasks containing an equivalent amount of dissolved glucose (50 mg/mL), BL21(DE3) cells expressing glucoamylase cultured in media containing starch showed no advantage in terms of final cell densities achieved.

### Demonstration of self-secreting enzyme-based fed-batch fermentation

**Fig. 8:**
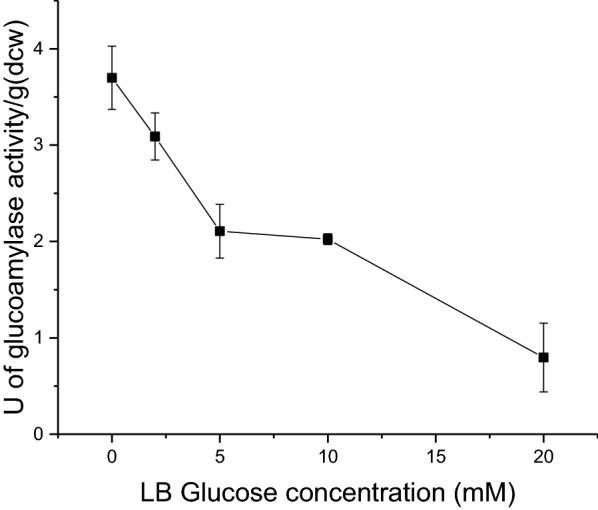
Characterisation of *PcstA* regulated *C. violaceum* glucoamylase secretion in LB media supplemented with 0–20 mM glucose. Extracellular glucoamylase activity of *E. coli* W3110 harbouring pQR1715 (BBa_K118011-CV2DsbA) cultured for 24 h at 37 °C, 250 rpm, in LB containing a range of glucose concentrations (0–20 mM). Activity expressed as units (defined as the disappearance of 1 mg/mL of starch/iodine complex per minute) per gram of dry cell weight and determined by the change in absorbance of starch/iodine complex at 600 nm. Error bars represent standard deviation, n = 3

*E. coli* BL21(DE3) is widely reported to be less susceptible to overflow metabolism than other strains such as W3110. A K12 compatible promoter was therefore used to investigate whether a glucose releasing W3110 amylolytic strain could achieve higher cell densities when cultured on starch as a carbon source compared with an equivalent amount of glucose. Expression from the glucose sensitive *PcstA* promoter was characterised by measuring extracellular glucoamylase activity following growth in LB containing varying concentrations of glucose. As the concentration of glucose increased, extracellular glucoamylase activity decreased, with a fourfold reduction in activity from 0 to 20 mM glucose (Fig. 8).

**Fig. 9:**
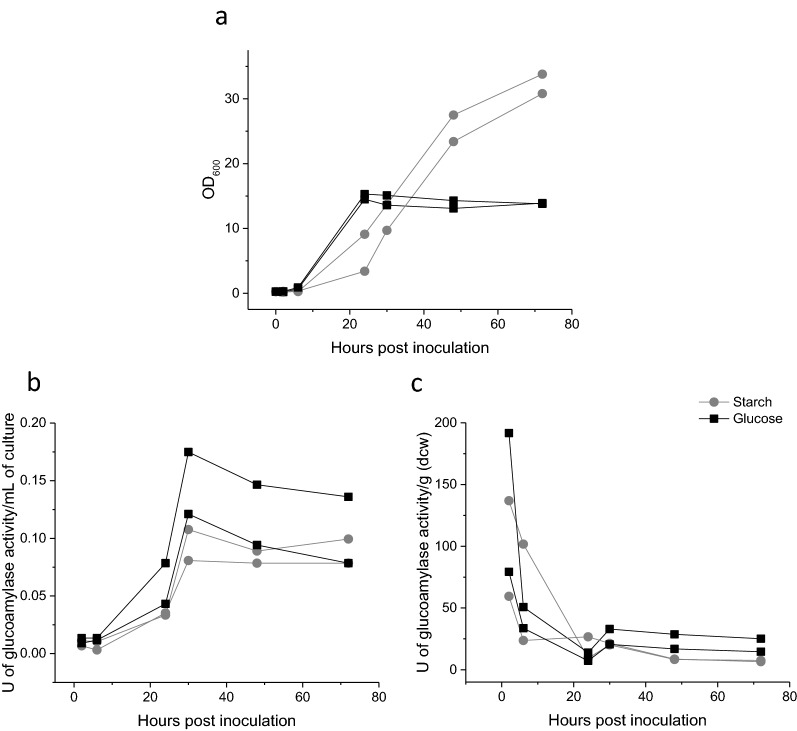
*PcstA* regulated *C. violaceum* glucoamylase expression and its influence on growth in MSM containing starch–agar layers. *E. coli* W3110 harbouring pQR1715 (BBa_K118011-CV2DsbA) cultured for 72 h at 30 °C, 180 rpm, in either glucose free MSM with starch–agar layers (circle) or MSM supplemented with 50 g/L glucose (square). **a** Growth curves for each condition in duplicate. **b** Glucoamylase activity in the extracellular media determined by amylase assay at 37 °C and expressed as units per mL of culture. **c** Glucoamylase activity in the extracellular media determined by amylase assay at 37 °C and expressed as units per gram of dry cell weight. Units defined as the disappearance of 1 mg/mL of starch/iodine complex per minute, determined by the change in absorbance of starch/iodine complex at 600 nm

The effect of self-regulated glucoamylase expression on growth was demonstrated using starch/agar layers (Additional file 1: Fig S4) and glucose free MSM in shake flasks. *E. coli* W3110 harbouring pQR1715 achieved substantially higher cell densities when cultured on starch (OD_600_ of approx. 30), compared with an equivalent amount of glucose (OD_600_ of approx. 15) (Fig. 9a). The associated extracellular glucoamylase activity was determined and expressed as both U per mL of culture and U per g(DCW), with one Unit defined as the disappearance of 1 mg of starch-iodine complex per minute. Surprisingly, throughout the culture period there was no difference in measured glucoamylase activity between starch and glucose media for either U per mL of culture (Fig. 9b) or U per g(DCW) (Fig. 9c). Both media types showed an initial increase in U per mL of culture for the first 30 h and then a subsequent plateau. When normalised to cell density activity decreased rapidly in the first 10 h and subsequently levelled off for the remainder of the culture period.

**Fig. 10:**
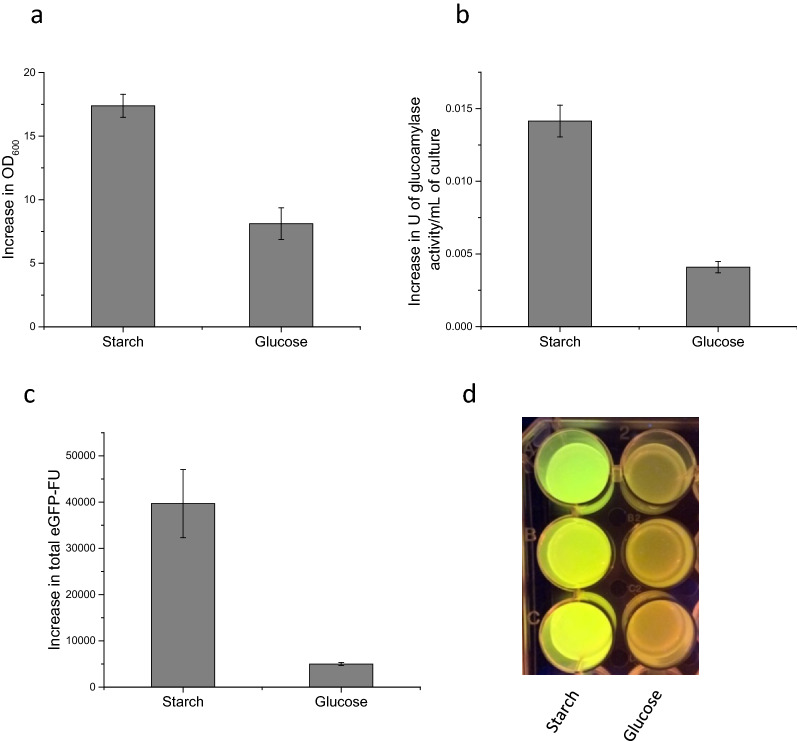
Self secreting enzyme-based fed-batch fermentation via simultaneous expression of *C. violaceum* glucoamylase and eGFP in *E. coli* W3110. *E. coli* W3110 harbouring pQR1720, cultured in flasks containing glucose free MSM with starch–agar layers or MSM supplemented with 50 g/L glucose, at 37 °C, 250 rpm for 48 h [induced with 0.4 mM IPTG at inoculation (initial OD_600_ of 0.25)]. **a** Increase in cell density (OD_600_) over 48 h of culture for each media type. **b** Increase in extracellular media glucoamylase activity over 48 h for each media type, expressed in units of glucoamylase activity per mL of culture, determined by amylase assay at 37 °C and expressed as units per mL of culture. Units defined as the disappearance of 1 mg/mL of starch/iodine complex per minute, determined by the change in absorbance of starch/iodine complex at 600 nm. **c** Increase in total eGFP fluorescence units over 48 h for each media type, measured from neat whole cell samples using an excitation wavelength of 483 nm and emission wavelength of 535 nm (Tecan infinite 200 pro). **d** Image of eGFP expression under blue light for each media composition, displayed in triplicate. Error bars represent standard deviation, n = 3

To determine whether the increase in cell density reflected an increase in recombinant protein yield, a vector was designed to simultaneously express *C. violaceum* glucoamylase and a recombinant protein of interest in the form of eGFP (pQR1720). Figure 10 shows this plasmid where the GFP and glucoamylase are constructed to be in an operon and expressed from a lac promoter. Cultures containing starch consistently achieved larger increases in both cell density (Fig. 10a) and eGFP fluorescence (Fig. 10c, d) from 0 to 48 h compared with the glucose equivalent; the first demonstration of enhanced recombinant protein yield directly resulting from self-secreting enzyme-based fed-batch fermentation.

## Discussion

The characterisation of two novel *D. geothermalis* and the *C. violaceum* glucoamylase enzymes within this study revealed both were more exo-acting than the previously characterised *T. tengcongensis* glucoamylase, with high performance anion exchange chromatography data displaying an increased accumulation of glucose relative to longer chain oligosaccharides when the former hydrolysed starch. Furthermore, the mass balance of starch hydrolysis and glucose accumulation was less negative for both the *D. geothermalis* and the *C. violaceum* glucoamylases, than for the *T. tengcongensis* glucoamylase, suggesting an accumulation of product other than glucose for the latter enzyme.

The increased ability of the *C. violaceum* glucoamylase to be secreted into the extracellular media is likely explained by the presence of a native signal sequence, in contrast with the *D. geothermalis* glucoamylase, which did not contain a native signal sequence. The replacement of the native signal sequence from the *C. violaceum* glucoamylase with the DsbA signal sequence saw a marginal but still significant improvement in secretion. The fact that the *D. geothermalis* glucoamylase did not contain a recognisable native signal sequence suggests it is not usually exported and therefore was probably not the best candidate enzyme for an extracellular application. Indeed, despite the addition of the DsbA signal sequence or the PelB signal sequence, no activity in the extracellular media was detected, indicating that *D. geothermalis* glucoamylase is not easily secreted through either the SRP or Sec pathways. Conversely, the *C. violaceum* glucoamylase is naturally secreted, and apparently capable of secretion by *E. coli* via both the native signal sequence and the exogenous DsbA signal sequence. There are two slightly different routes that the standard *E. coli* signal sequences are recognised and processed. These are the SEC and SRP pathways. The PelB signal sequence is processed via the SEC pathway and the DsbA sequence is processed via the SRP pathway. In some cases the SRP signal sequence helps secretion of certain proteins which are not efficiently secreted by the SEC pathway. But in the case of *D. geothermalis* neither the SRP or the SEC pathway allowed secretion.

Secreted *C. violaceum* glucoamylase via either signal peptide showed considerable glucose releasing amylolytic activity in the extracellular media, enough to allow expression strains to degrade starch contained within the medium during growth. Furthermore, *E. coli* BL21(DE3) expressing and secreting *C. violaceum* glucoamylase was able to utilise starch as a sole carbon source, which to our knowledge is the first example of an engineered *E. coli* strain converting starch directly to glucose for the purpose of enhancing growth. To mimic fed-batch fermentation using the slow release of utilisable carbon, a combination of defined media (glucose free MSM) and starch-agar layers were developed alongside the *C. violaceum* glucoamylase *E. coli* W3110 expression strains. The secretion of *C. violaceum* glucoamylase was designed to be negatively regulated by its own hydrolysis product, theoretically maintaining a constant glucose concentration in the media throughout the culture, irrespective of cell density. A Cyclic Adenosine Monophosphate (cAMP) sensitive promoter (*PcstA*) was selected and characterised. *PcstA* itself, derived from *E. coli* JM109, is ordinarily involved in the regulation of carbon starvation response genes (*cstA*) which are up regulated during glucose starvation [23]. The mechanism behind this is based on the ability of cAMP to induce conformational change in the catabolite gene activator protein (CAP), enhancing its binding to CAP-dependent promoters [24], and promoting expression. In the absence of glucose, levels of intracellular cAMP are increased [25], leading to the binding of CAP to the promotor and subsequent transcription of target genes. In high glucose concentrations, intracellular cAMP is reduced, leading to a reduction in bound CAP, and a subsequent reduction in gene transcription. *E. coli* W3110 expressing *C. violaceum* glucoamylase under the regulation of *PcstA* was capable of achieving densities approximately twice that of cultures grown in an equivalent amount of glucose; demonstrating increased cell densities as a direct result of self-regulated enzyme-based fed-batch fermentation.

The main caveat to this system however, is that, for shake flasks at least, the advantages may be specific to K12 strains, as BL21(DE3) appeared not to benefit from the slow release of glucose from starch, presumably because BL21(DE3), being a B strain of *E. coli*, does not demonstrate any detrimental effects in terms of growth from high concentrations of glucose in the first place. This is likely a result of the B strain’s, BL21(DE3), ability to employ a glyoxylate shunt, reducing the accumulation of acetate and minimising the detrimental effects of overflow metabolism [26–30].

The data for the host strains with no plasmid versus the same hosts containing plasmids secreting the *C. violaceum* glucoamylase or an alpha amylase in starch containing medium shows that in both cases the growth rate and final OD are significantly greater in the cells that have the plasmid. This shows that there is no ‘burden’ given by carriage and expression of the secreted enzymes in the system we are describing here. The data for the *E. coli* with and without the plasmid expressing *C. violaceum* glucoamylase in Fig. 6 and in Additional file 1: Figure S3 shows that an alpha-amylase expressing plasmid gives significantly higher growth rates and final OD compared to plasmid free cells in rich media with starch.

**Fig. 11:**
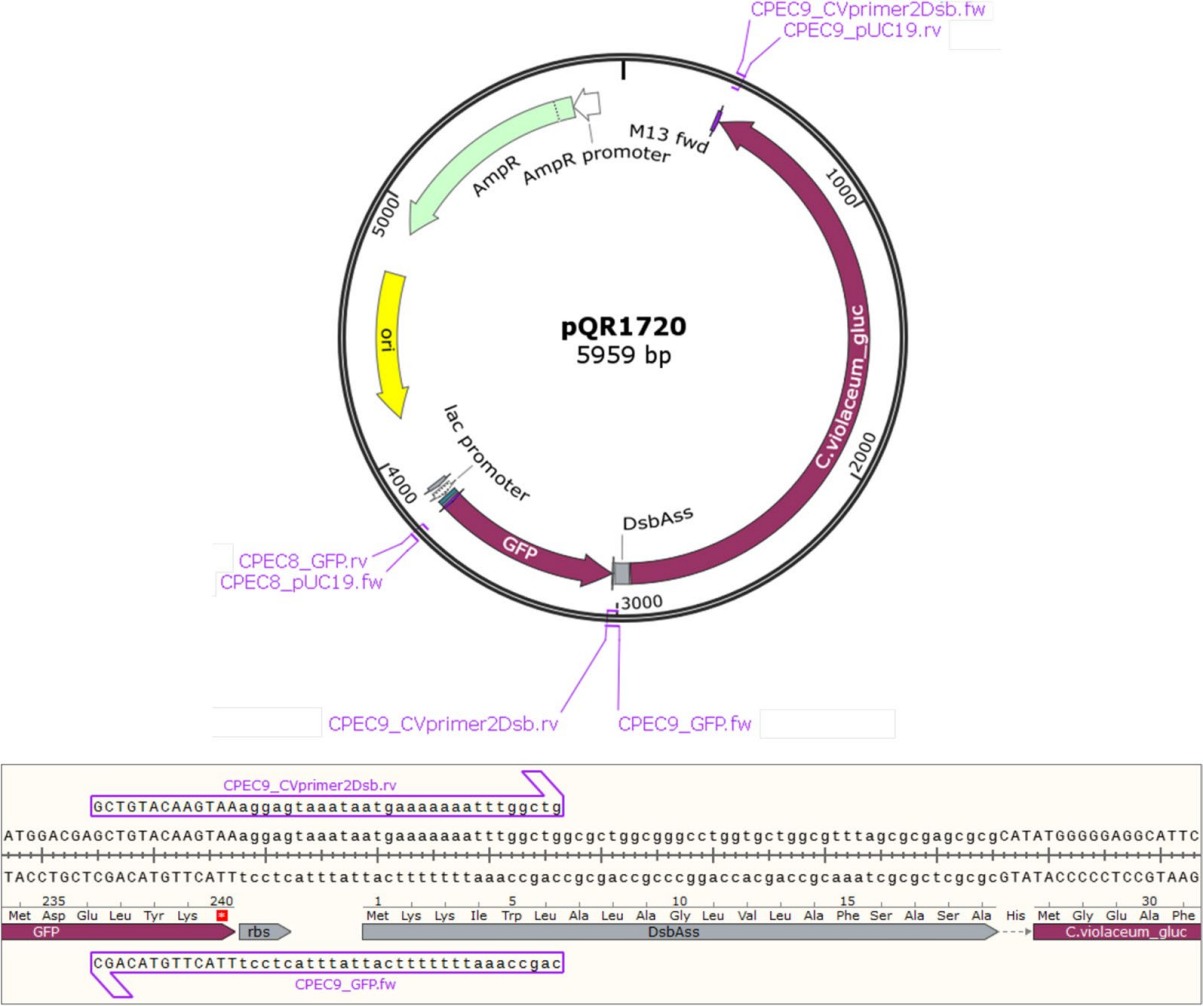
Vector designed for simultaneous expression of eGFP and *C. violaceum* glucoamylase. Vector map of pQR1720, constructed using multiple insert CPEC following insert and vector PCR reactions using the primer pairs indicated. Insert 1 (eGFP) amplified using CPEC8_GFP.rv and CPEC9_GFP.fw primers, with pQR1344 as the template, insert 2 (*C. violaceum* glucoamylase with attached DsbA signal sequence) amplified using CPEC9_CVprimer2Dsb.rv and CPEC9_CVprimer2Dsb.fw primers, with pQR1712 as the template, and the vector backbone amplified using CPEC9_pUC19.rv and CPEC8_pUC19.fw primers, with pUC19 as the template

Finally, in an attempt to use the enhanced cell mass as a platform for recombinant protein expression, simultaneous expression of both an amylolytic enzyme and a recombinant protein of interest was investigated. Rather than using two separate vectors, both genes were expressed from the same vector. The rationale was that this would eliminate compatibility issues, reduce the metabolic burden associated with multiple antibiotic resistance genes, and simplify the transformation method. In addition it would also provide a basis for a versatile system whereby a plasmid already containing a self-regulated amylolytic enzyme could be designed to contain a multiple cloning site for the insertion of an additional gene of interest, similar in concept to current commercially available vectors such as pETDuet™ (Novagen) or pACYCDuet™ (Novagen), but specifically used for high density growth using starch as a substrate. The construction of pQR1720 which included an operon containing eGFP immediately followed by the *C. violaceum* glucoamylase (Fig. 11), regulated by the *lac* promoter, successfully demonstrated a recombinant protein expression system using starch as a sole carbon source. pQR1720 is based on pUC19 and is a very high copy number plasmid with approximately 500–700 copies per cell. The eGFP in pQR1720 is expressed from the lac promoter which is considerably weaker than the T7 promoter in the pET constructs. The previous constructs in pET are in a low/medium copy number backbone giving 15–20 copies per cell. This is a 25- to 45-fold difference in gene dosage between pUC and pET (pUC $\gg$ pET) which evens out the influence of the weaker promoter. So although the promoter pPlac is weaker than the T7 promoter, the large increase in copy number makes up for this.

Furthermore, the yield of recombinant protein (eGFP) was greater in cultures containing starch than in cultures containing an equivalent amount of glucose; the first example of enhanced recombinant protein expression resulting directly from a cell engineered approach to enzyme-based fed-batch fermentation.

## Conclusion

This study provides an innovative solution to a fundamental problem associated with microbial fermentation, achieving high cell densities and increased recombinant protein yields in batch culture. By combining the principles of enzyme-based fed-batch fermentation with engineered amylolytic *E. coli* strains, a proof of concept has been demonstrated which has the potential to provide an alternative to current traditional fed-batch methods. More broadly, this work highlights the capability of cell engineering to enhance bioprocessing, and provides a platform for further projects looking to combine this with other cell engineering approaches to improve overall bioprocessing efficiencies.

## Methods

### Strains and plasmids

*E. coli* strains used in this study were W3110 (F^−^ λ^−^ *rph-1*, *Inv*(*rrnD*, *rrnE*)) [72], and BL21(DE3) [73] (F^−^ *ompT*, *gal*, *dcm*, *lon*, *hsdS*_*B*_(*r*_*B*_^*−*^*m*_*B*_^*−*^) l(DE3 [*lacI* *PlacUV5*-*T7p07* *ind1* *sam7* *nin5*]) (Novagen). HST08 (Stellar™ chemically competent, Clonetech) and TOP10 (chemically competent, Thermo Fisher Scientific) were used for sub-cloning experiments. All vectors, primers and oligonucleotides were designed using SnapGene (version 1.1.3) software. Oligonucleotides were synthesised and supplied by Eurofins Genomics. For use in PCR, lyophilised oligonucleotides were re-suspended in miliQ water and diluted to a concentration of 10 µM. For use in ligation, equimolar concentrations of complementary oligonucleotides were mixed, heated to 98 °C and allowed to cool to room temperature. Vectors were constructed using traditional restriction digestion and ligation or by circular polymerase extension cloning. Confirmation of correct construction was carried out by restriction analysis and sequencing (Eurofins Genomics). All strains were maintained and propagated using selective LB agar plates, supplemented with kanamycin (20 µg/mL), ampicillin (50 µg/mL) or chloramphenicol (30 µg/mL) (Table 1).

**Table 1:** Constructs used within this study

Plasmid	Description	References
pET29a(+)	T7 promoter, Kan^r^, ability for C-terminal 6-His tag	Novagen
pQR1344	pET29a containing eGFP, T7 promoter, Kan^r^	D. Dobrijevic (unpublished)
pQR1703	pET29a containing *D. geothermalis* glucoamylase, T7 promoter, Kan^r^	This study
pQR1704	pET29a containing *T. tengcongensis* glucoamylase, T7 promoter, Kan^r^	This study
pQR1705	pET29a containing *C. violaceum* glucoamylase, T7 promoter, Kan^r^	This study
pET28a(+)	T7 promoter, Kan^r^, ability for N-terminal 6-His tag	Novagen
pQR1706	pET28a containing *D. geothermalis* glucoamylase, T7 promoter, Kan^r^	This study
pQR1707	pET28a containing *T. tengcongensis* glucoamylase, T7 promoter, Kan^r^	This study
pQR1708	pET28a containing *C. violaceum* glucoamylase, T7 promoter, Kan^r^	This study
pQR1709	pQR1703 with DsbA signal sequence, T7 promoter, Kan^r^	This study
pQR1710	pQR1703 with PelB signal sequence, T7 promoter, Kan^r^	This study
pQR1711	pQR1705 lacking native signal sequence, T7 promoter, Kan^r^	This study
pQR1712	pQR1711 with DsbA signal sequence, T7 promoter, Kan^r^	This study
BBa_K118011	pSB1C3 containing *PcstA*, Cam^r^	iGEM 2016 Distribution Kit
pQR1715	BBa_K118011 containing *C. violaceum* glucoamylase with attached DsbA signal sequence, *PcstA*, Cam^r^	This study
pUC19	*lac* promoter, Amp^r^	Yanisch-Perron et al. [31]
pQR1720	pUC19 containing eGFP followed by *C. violaceum* glucoamylase with DsbA signal sequence, *lac* promoter, Amp^r^	This study

### Chromosomal DNA preparation

*Thermoanaerobacter tengcongensis* was supplied lyophilised by The Leibniz Institute DSMZ (DSM number 15242 *Caldanaerobacter subterraneus* subsp. *tengcongesis*), and was re-suspended in 1 mL of 20% (w/v) glycerol. *Chromobacterium violaceum* and *Deinococcus geothermalis* were supplied by J. Ward as glycerol stocks. Chromosomal DNA was prepared by diluting the glycerol stocks 1 in 10 with sterile water. Lysozyme was added to this dilution to give a final concentration of 50 µg/mL and the mixture was incubated at 37 °C for 15 min. SDS was then added to give a final concentration of 1% (v/v), turning the mixture clear and viscous. 250 µL of this solution was combined with a vortexed mixture of P2 (250 µL) and N3 (350 µL) reagents (QIAprep Miniprep Kit, Qiagen) and centrifuged for 10 min at 17,000×*g*. The supernatant was loaded onto a spin column (QIAprep Miniprep Kit, Qiagen) with the standard miniprep protocol followed from this point onwards. The genomic DNA was eluted with 100 µL EB buffer, and used directly for PCR. Genomic DNA from *D. geothermalis* was provided by Dr Maria Bawn.

### Plasmid construction

Glucoamylase genes from *D. geothermalis*, *T. tengcongensis*, and *C. violaecium* were amplified using PCR under standard conditions [Phusion® High-Fidelity PCR master mix with HF Buffer (New England Biolabs)]. With an annealing temperature gradient of 10 °C (5 °C either side of the lowest primer T_m_) each PCR produced bands of the expected size; *D. geothermalis* (2396 bp), *T. tengcongensis* (2111 bp), and *C. violaecium* (2624 bp). Following gel extraction and purification (QIAquick Gel Extraction Kit, Qiagen), DNA bands were digested with NdeI and XhoI (New England Biolabs) and ligated (T4 DNA ligase, New England Biolabs) to purified NdeI/XhoI digested pET29a or pET28a backbones (Novagen). The resultant constructs, pQR1703, pQR1704, pQR1705 and pQR1706, pQR1707, pQR1708 respectively, were confirmed through restriction digestion analysis and sequencing (Eurofins Genomics).

The addition of signal peptides to the *D. geothermalis* glucoamylase was carried out by annealing complementary oligo nucleotides corresponding to DsbA and PelB signal sequences (synthesised by Eurofins Genomics), with the 5′ end of the sense strand and the 3′ end of the antisense strand staggered to form a join compatible with an XbaI restriction site, and the 3′ end of the sense strand and the 5′ end of the antisense staggered for compatibility with an NdeI restriction site. A ribosome binding site and a spacer region were included between the XbaI restriction site and the start codon. Following XbaI/NdeI digestion of pQR1703 the annealed oligo nucleotides were ligated to the backbone forming pQR1703-DsbA and pQR1703-PelB, designated pQR1709 and pQR1710 respectively. The addition of DsbA signal sequence to the *C. violaceum* glucoamylase first required the removal of the native sequence, using an alternative forward primer which excluded the native signal sequence but contained an NdeI site (including a start codon) in the overhanging region. The truncated gene was then cloned into pET29a forming pQR1711, which itself was used as the backbone for the insertion of annealed complementary oligo nucleotides, creating pQR1711-DsbA, designated pQR1712.

Plasmids pQR1715 and pQR1720 were constructed using Circular Polymerase Extension Cloning (CPEC) [32]. Reactions were carried out in total volumes of 50 µL containing 25 µL Phusion® High-Fidelity PCR master mix with HF Buffer, purified vector (200 ng) combined with purified insert at a 1:1 molar ratio. The thermo-cycling conditions for CPEC reactions used an initial denaturation period of 30 s at 98 °C, followed by cycles of denaturation (98 °C for 15 s), annealing (55 °C for 30 s), and extension (72 °C for 25 s per 1000 base pairs) and a final extension of 72 °C for 5 min. One cycle was performed for single insert CPEC (pQR1715), but for multiple inserts (pQR1720) this was increased to 20 cycles. CPEC reaction product was used directly for transformation (4 µL CPEC product in 50 µL competent cells; Table 2).

**Table 2:**
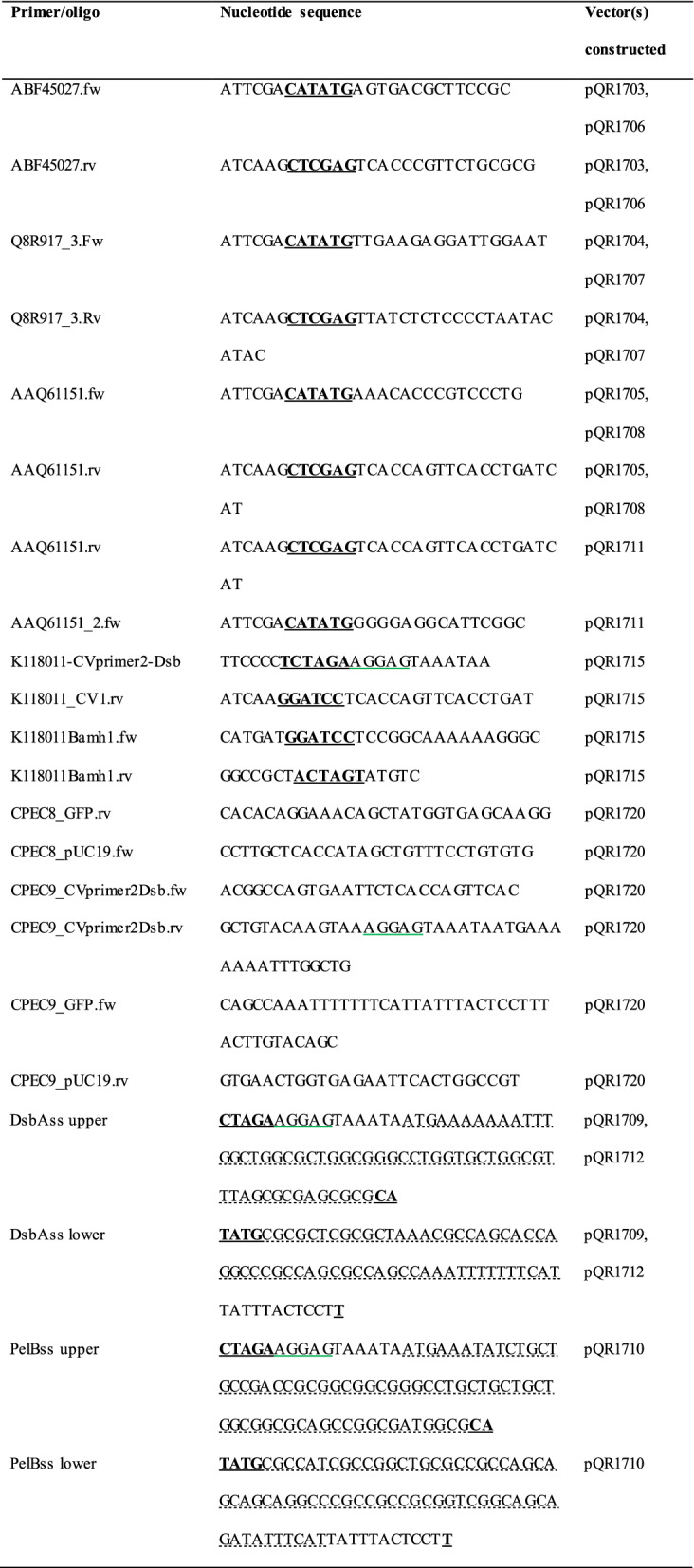
Nucleotide sequences of primers and oligonucleotides used in vector construction

Restriction sites indicated by underlined bold (**CATATG** = NdeI, **CTCGAG** = XhoI, **TCTAGA** = XbaI, **GGATCC** = BamHI, **ACTAGT** = SpeI) Signal sequence dashed underlined, and ribosome binding site green underlined. The DsbAss upper and DsbAss lower are long oligos designed to anneal to one another and create a double stranded DNA fragment with an XbaI sticky end and an NdeI site at the other end. The PelB upper and lower anneal to also have XbaI and NdeI sticky ends. There is a ribosome binding site, , and a spacer region, TAAATA, included between the XbaI restriction site and the start codon

### Media and shake flask composition

Miller’s LB Broth (Sigma Aldrich), containing 10 g/L Tryptone, 10 g/L NaCl and 5 g/L yeast extract, was prepared by dissolving 25 g in 1 L of water. Terrific broth (Sigma Aldrich), containing 12 g/L Tryptone, 24 g/L yeast extract, 9.4 g/L K_2_HPO_4_, and 2.2 g/L KH_2_PO_4_, was prepared by dissolving 47.6 g plus an additional 8 mL of glycerol into 1 L of water. Both were autoclaved at 121 °C for 15 min.

Glucose free mineral salt medium (MSM) was adapted from Krause et al. [9] and was prepared by dissolving (per L of water) 2.0 g Na_2_SO_4_, 6.12 g (NH_4_)_2_SO_4_, 0.50 g NH_4_Cl, 14.60 g K_2_HPO_4_, 3.60 g NaH_2_PO_4_·H_2_O, 1.00 g (NH_4_)_2_-*H*-citrate, 3 mM MgSO_4_, 0.1 g thiamine hydrochloride, and 2 mL of SM6 trace element solution (composed of 104 g/L Citric acid, 5.22 g/L CaCl_2_·2H_2_O, 2.06 g/L ZnSO_4_·7H_2_O, 2.72 g/L MnSO_4_·4H_2_O, 0.81 g/L CuSO_4_·5H_2_O, 0.42 g/L CoSO_4_·7H_2_O, 10.06 g/L FeCl_3_·6H_2_O, 0.03 g/L H_3_BO_3_, 0.02 g/L Na_2_MoO_4_·2H_2_O). The solution was filter sterilised using 0.22 µm Millipore Express™ PLUS stericup® with a vacuum pump. MSM was prepared in three formats, either glucose free MSM, MSM supplemented with 2.5 mg/mL glucose (Sigma), or MSM supplemented with 2.5 mg/mL starch (soluble potato starch, Sigma). To assist with dissolving starch the latter required pre-autoclaved water containing enough starch for a final concentration of 2.5 mg/mL prior to addition of the other constituents and filter sterilisation.

Starch-agar layers were prepared within 500 mL baffled shake flasks, and were used in conjunction with glucose free MSM media. Firstly, a 50 mL solution of 50 mg/mL starch and 2 mg/mL agar was added to the flask and autoclaved at 121 °C for 15 min. Once cooled, 50 mL of pre-autoclaved 50 mg/mL agar was poured on top of the original layer and allowed to cool. Finally, 50 mL of glucose free MSM was added to the flask, remaining above the layers (Additional file 1: Figure S4).

### Growth and expression studies

All shake flasks used for growth and expression studies were inoculated with either a 1 in 20 dilution from an overnight culture, or inoculated to an initial OD_600_ of 0.1 or 0.25. Overnight cultures were generally 5 mL LB in a 50 mL falcon tube (containing appropriate antibiotic) seeded from a glycerol stock, and grown at 37 °C at 250 rpm. Unless otherwise stated, 500 mL baffled conical shake flasks were used, containing 50 mL of selected media, and in some cases the starch/agar layers described previously. In addition to media, an antibiotic, if needed, was supplied at the following concentrations: kanamycin (20 µg/mL); ampicillin (50 µg/mL); chloramphenicol (30 µg/mL). For induction of all inducible promoters used in this study, 0.4 mM IPTG was added when the culture had reached an OD_600_ of 0.6–0.8, or at inoculation, for growth experiments involving amylolytic strains. Shake flask cultures were carried out at varying temperatures and durations depending on the nature of the experiment (see individual experiment for detail), but all were performed in a standard orbital incubated shaker (innova™ 4330, New Brunswick Scientific) at either 250 or 180 rpm.

Cell free extract—samples (1 mL) of culture were centrifuged at 17,000×*g* for 5 min. The resultant pellet was re-suspended in 300 μL phosphate buffered saline (PBS) (pH7.4) and sonicated (3 cycles of 20 s on, 20 s off with an amplitude of 10 microns, MSE Soniprep 150). The ensuing whole cell fraction was further centrifuged at 17,000×*g* for 5 min and the supernatant collected as the clarified lysate.

Extracellular fraction—for the determination of secreted amylolytic enzyme within the extracellular media, 1 mL of culture was centrifuged at 17,000×*g* for 5 min with the resultant supernatant collected as the extracellular media fraction. To quantify activity from this fraction, samples were directly added to the amylase assay.

### Amylolytic activity assays

Amylolytic activity was determined by measuring the rate of degradation of starch–iodine complex, a method adjusted from Blanchin-Roland and Masson [33], or the resulting accumulation of glucose via high performance anion exchange chromatography with pulsed amperometric detection (HPAEC-PAD) analysis. Unless otherwise stated amylolytic activity assays were set up using a 50 µL sample of extracellular media or cell free extract made up to 0.5 mL with 15 mM sodium phosphate buffer (pH5.8), and incubated at 37 °C with 0.5 mL starch solution [5 mg/mL starch in 15 mM sodium phosphate buffer (pH5.8)]. Subsequent 50 µL samples were then taken at numerous time points and added to 1 mL of potassium iodide/iodine solution [freshly prepared by adding 200 μL 2.2% I_2_/4.4% KI (w/v) into 100 mL of 2% (w/v) KI solution] with the corresponding decrease in absorbance at 600 nm measured. One unit of enzyme activity was defined as the disappearance of 1 mg/mL of starch-iodine complex per minute at 37 °C. In addition, samples were also taken for HPAEC-PAD analysis, for the detection and quantitation of mono- and small chain oligosaccharides resulting from the amylolytic hydrolysis of starch. Detection was carried out by injecting 25 µL samples into a Reagent-Free Ion Chromatography System (ICS 5000+, Dionex) equipped with a Dionex CarboPac™ PA100 anion exchange column (2 × 250 mm) fitted with a Dionex CarboPac™ PA100 guard column (2 × 50 mm), and an electrochemical detector system. Elution was carried out using a linear gradient of 100 mM NaOH at 0 min, to 60 mM NaOH and 400 mM NaOAc at 18 min, followed by a step of 40 mM NaOH and 600 mM NaOAc for a further 2 min. The column was re-equilibrated for 5 min with 100 mM NaOH. The flow rate remained at 0.25 mL min^−1^ with a system pressure of approximately 2500 psi. Data collection and analysis was performed with Chromeleon software version 7.

Starch degradation within culture media was determined using an adaptation of the amylolytic activity assay described previously, whereby a 50 µL sample of clarified culture media was added directly to 1 mL of potassium iodide/iodine solution [freshly prepared by adding 200 μL 2.2% I_2_/4.4% KI (w/v) into 100 mL of 2% (w/v) KI solution]. The absorbance was measured against a potassium iodide/iodine solution blank at 600 nm, with a standard curve prepared to determine concentration. For concentrations of starch beyond the linear range of the spectrophotometer the sample of clarified culture media was diluted appropriately and added to the potassium iodide/iodine solution in the same ratio.

Fluorescence measurements—fluorescence intensity of eGFP from samples of neat cell culture dispensed in either 24 or 96 microwell plates, were measured using excitation and emission wavelengths of 483 nm and 535 nm respectively (Tecan Infinite M200 Pro). Gain was optimised for each experiment conducted. Fluorescence units were displayed either irrespective of cell density, or were normalised to OD600 depending on the specific experiment conducted.

## Supplementary Information

**Additional file 1: Figure S1.** Alignment of the glucoamylases from *Thermoanaerobacter tengcongensis* MB4, *Deinococcus geothermalis* and *Chromobacterium violaecum*. **Figure S2.** Crude (whole cell) and clarified lysates analysed on SDS page for pQR1706, pQR1707 and pQR1708. **Figure S3.** Growth curves of α-amylase secreting *E. coli* compared to plasmid free *E. coli* cultured in commercially available high cell density media. **Figure S4.** Design of starch-agar layer system for sufficient provision of carbon for high cell density applications.

## Data Availability

Data generated or analysed during this study are included in this published article. Any further data analysed for this study are available from the corresponding author.
