# Supplementary material for: A cell engineering approach to enzyme-based fed-batch fermentation
Source: Microb Cell Fact. 2021 Jul 24;20:146. doi: 10.1186/s12934-021-01634-y (PMC8310608; doi:10.1186/s12934-021-01634-y)
Supplement: Supplementary file 1 — Additional file 1: Figure S1. Alignment of the glucoamylases from Thermoanaerobacter tengcongensis MB4, Deinococcus geothermalis and Chromobacterium violaecum. Figure S2. Crude (whole cell) and clarified lysates analysed on SDS page for pQR1706, pQR1707 and pQR1708. Figure S3. Growth curves of α-amylase secreting E. coli compared to plasmid free E. coli cultured in commercially available high cell density media. Figure S4. Design of starch-agar layer system for sufficient provision of carbon for high cell density applications. [file 12934_2021_1634_MOESM1_ESM.docx]

Additional Figures for: **A Cell Engineering Approach to Enzyme-Based Fed-Batch Fermentation**

Michael Sibley and John M Ward*

Department of Biochemical Engineering, UCL, Gower Street, London, WC1E 6BT

D. MSDASAQNPPEQLLATPGSPELISPAQGLAPGAPGLPPTWASSDKDFVTTALGGASRLWA 60

T. -------------CSDVSYVKVEHLDKTEASQGPGERDTWATAQKQGIGTANNDVSKVWF 47

C. ---------------------------GEAFGSPGGAPVRGPAAKSFVGTAVNSPSRVYF 33

* .** . . : *. : ** .. *:::

D. TGGHGMLNEVYWPSTGQPQIRDLTFYLVGAAGWVDLR-RVRRYQLS--TPKPYLPLPTLL 117

T. TLAQGALSEIYYPTIDRANSKFLKFIVTDGKTFVADETTDTVSKVEKINNRSLAYRLVNI 107

C. TGYRGIVSEVYYPLLDTPESVDLQFLVGDAGRTFVDEEKRQAYSASQSDKRTMSWVASTG 93

* :* :.*:*:* . : * * : .. . . . . :

D. HQGDDYQLMLEVLPDPHRDVLLIRYAL-------SGPYRLAIVLAPHLTSTGHDNAAWVE 170

T. DKRGRYKITKEIFTDPRRNSVVMKVRFEALKGK-MEDYKLYLVYDPHISNQGADNEGYVV 166

C. NAGHNWQISKRIFADPNRDALVQRVTFTALNGHTVGDFNLYALFKPYLDNSGSGNSAQTV 153

. ::: .:: **.*: :: : : :.* : *:: . * .* . .

D. G----QHLLAVSGNRALALLSSSR------MEHLSAGYVGVSDGWQDLHQHG--RLTWSY 218

T. KANGEYGFMACRNNVYSALMTDAKWGSY------SVGYNGVNDPVSDLKKNK--KMTYKF 218

C. AVAGGYALAASRNSRASALMASLPWKTVNGQAMLSSGFVGQSDGWTDLIGSGDNTMNWTY 213

: * .. **::. * *: * .* ** :.:.:

D. ERAENGNVALSAELQDA-------SGLLALGFAENVTGAQGLARASLAEGDEPARRAFLY 271

T. DRA-KGNIIEGIEIDLR----DKTEFKTVLSFGESEEEALKTALSTLKDSYDRMLGIYIA 273

C. GSATNGNVAQTGWLDLGDPTATSVSFDMVLAFGKNQGDAVNAAAAALGSDLSAAQQQYDN 273

* :**: :: . .*.*.:. * * ::* .. . :

D. AWEAWGSALKLGGPSPELEAEALLSATVLKVHEDRTYPGALVASLSIPWGDST--DTLGG 329

T. EWNKYCDGLKNFGGE--ADELYYTSLMFLKASEDKTNKGAFIASLSIPWGEGQGDENKGG 331

C. AWHAYAAGLSNQGGL--ADDRYYLAAMTLKTMQDKS-NGAMIAGIGTPWGETQGDTNQGG 330

*. : .*. * : : **. :*:: **::*.:. ***: . **

D. YHLVWPRDATLAAFALLACNQREDARRVLAWFIA--------------------NQQPDG 369

T. YHLVWARDLYHIANAFIAAKDIDSANRALDFLAM--------------------VVEKNG 371

C. YHLVWPRDLFKFANALYTAGDAATAASVVNYLFNTLQQTSDCGAAEYNAPGCAAGYSRVG 390

***** ** * *: :. : * .: :: . *

D. HWLQNYYPDGQDFWHGVQLDETAFPVLLAAKLREEGEPELEGTRDMVRRALAFVARTGPT 429

T. FMPQNTWINGDPYWNGIQMDEQADPIILAYHLKRYDL-----YEKLVKPLADFIVRVGPK 426

C. RFPQNAWISGWPYWQGTQMDEQAMPILLAWRLGPAVS---NPLWPKIKQTADYIVATGPW 447

** : .* :*:* *:** * *::** :* :: ::. .**

D. SDQDRWEENQGVNPFTLAVAIAALVAGSGWLEED----ERHYALSLADDWNERLESLCYV 485

T. TGQERWEEAGGYSPATMAAEVAGLVCAADIAKQNKDMERAKKYLETADKWQELIDKLTYT 486

C. SYQERWEENAGYSPSTIAAEIAGLVAAADIARANGDDASAGRYLSAADYWQQNVATWTYT 507

: *:**** * .* *:*. :*.**..:. . : *. ** *:: : . *.

D. TGTPLCRELGVEGYYVRLAPPDRDGTLTGQVTLQ-------------NRQGKTVEAAALV 532

T. TKGP----YGNGQYYIRIAGL---------------PDPDADFLISIANGGGVYDQKEIV 527

C. TSGS----FGNGSYYVRINPANRSGSGADRASFNPAAGPDTPQTLTVKNGGGSHDARRVV 563

* * **:*: . * : :*

D. SLDFSYLPRLGLRSALDPRIRDTVKVVDQLLAQKT-------PTGIFYHRYNGDGYGEHE 585

T. DPSFLELVRLGVKAYDDPKILNTISVVDSLLKVNT-------PKGPSWYRYNHDGYGEPA 580

C. DGGFLELVRMGVKRADDPTIVNTMAVFDSVLGQNLALPGAPALPANAWFRYNFDGYGEHN 623

. .* * *:*:: ** * :*: *.*.:* : . :.*** *****

D. DGAPYDGSGMGRLWPLLSGERGHLALQAGE---DATVYLNSLLRCSSPGGLLPEQVWDGP 642

T. KGELYHGKGKGRLWPLLTGERGMYEIAAGK---KADDYLEYMRNFANEGFVLSEQIWEDT 637

C. DGRDFDGTGAGRLWPIFTAERGMYEIARQGAGSAGQPYFATLKLLATPEGMLPEQVWSNS 683

.* :.*.* *****:::.*** : . *: : :. :* **:*..

D. PLP--------ERGLFPGRPSGSAMPLLWAHAEFLKLLHTAQTGRPAELLREVEERYRQP 694

T. -----------------GLPTDSASPLNWAHAEYVVLFASNIEGKVVDMPQIVYKRYVLG 680

C. ATLPDGWAVTTPAGYRPGSATKSMGPLNWAMGEYISLLASIGAGRVVDIPQVVCARYNNC 743

* : * ** ** .*:: *: : *: .:: : * **

D. LPAQARHWRPAA--PVPELEPGLLLLIEDDKPFLLHYGFDGWQNPQDRPALRLPFGLWGV 752

T. ER---------------------------------------------------------- 682

C. RAAPKSGEVAVAINASATTQWGQQVYVTGNA-----RALGNWNTDLGIPLDAAAYPSWKN 798

D. --TFSPGELREHHTLDFTRKLAVGWEGQDHHIRLHEGAPK-ASLTAQNG- 798

T. -------------------------------------------------- 682

C. GANLPAGQQIAYKYYRKNADGSVSWENLAGNRSLQTPASGSLSLNDQVNW 848

**Figure S1. Alignment of the glucoamylases from *Thermoanaerobacter tengcongensis* MB4, *Deinococcus geothermalis* and *Chromobacterium violaecum.***

Clustal Omega alignment using the signal sequence free glucoamylase sequences of *Chromobacterium violaceum,* C*,* and *Caldanaerobacter subterraneus* subsp. *tengcongensis* (also known as *Thermoanaerobacter tengcongensis,* T*.* The native glucoamylase from *Deinococcus geothermalis*, D, has no signal sequence.


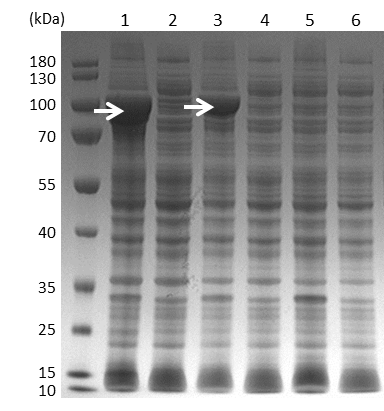


**Fig S2 Crude (whole cell) and clarified lysates analysed on SDS page for pQR1706, pQR1707 and pQR1708**.

*D. geothermalis* glucoamylase, pQR1706, whole cell lysate (lane 1) and clarified lysate (lane 2), *C. violaceum* glucoamylase, pQR1708, whole cell lysate (lane 3) and clarified lysate (lane 4), *T. tengcongensis* glucoamylase, pQR1707, whole cell lysate (lane 5) and clarified lysate (lane 6). Arrows indicate the presence of *D. geothermalis* glucoamylase (87.5 kDa) and *C. violaceum* glucoamylase (92.7 kDa) within the crude lysates (lanes 1 and 3 respectively). *E. coli* BL21 (DE3) harbouring either pQR1706, pQR1707, or pQR1708, induced (0.4mM IPTG) at OD_600_ of 0.8 and grown in TB for 24 hours at 25°C.

Fig S3 Growth curves of α-amylase secreting *E. coli* compared to plasmid free *E. coli* cultured in commercially available high cell density media

*E. coli* W3110 (black) and W3110 harbouring pQR187 (grey) grown as per the EnPresso® growth system protocol, with the latter induced from inoculation (0.4 mM IPTG) and excluding the additional reagent A. pQR187 is a derivative of pQR126 (French et al, 1996; Pierce et al, 2002) containing a PstI/EcoRI 200 base pair *cer* fragment of pKS450 (Summers and Sherratt, 1984). The *cer* fragment impart segregational stability to the plasmid (French and Ward, 1995) so that antibiotics are not needed to ensure stable carriage of the plasmid by the *E. coli* strain.

French C., Keshavarz-Moore., E., and Ward J. M. (1996) Development of a simple method for the recovery of recombinant proteins from the Escherichia coli periplasm. Enzyme Microbial Tech. 19, 332-338

J.J. Pierce, S.C. Robinson, J.M. Ward, E.Keshavarz-Moore, P. Dunnill. (2002) A comparison of the process issues in expressing the same recombinant enzyme periplasmically in *E.coli* and extracellularly in *S.lividans*. J. Biotech. 92, 205-215

French C., and Ward J. M. (1995) Improved production and stability of E. coli recombinants expressing transketolase for large scale biotransformations. Biotech. Lett. 17, 247-252

Summers DK, Sherratt DJ. (1984) Multimerization of high copy number plasmids causes instability: CoIE1 encodes a determinant essential for plasmid monomerization and stability. Cell. 36:1097-103.


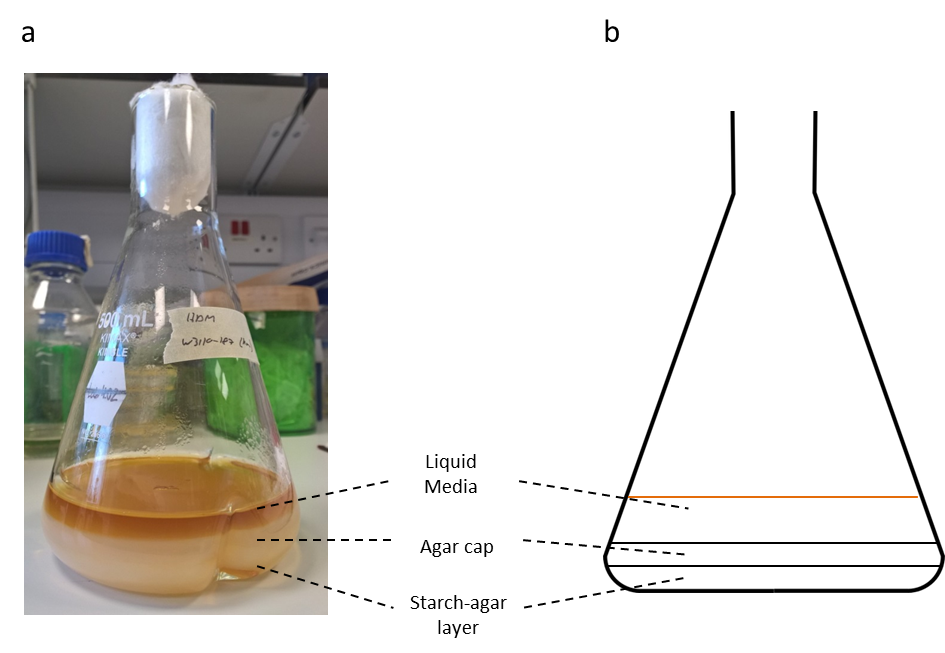


c

**Figure S4.** **Design of starch-agar layer system for sufficient provision of carbon for high cell density applications**

Photograph (a) and diagram (b) of the starch-agar layer design containing as a bottom layer 50 mL of 5% (w/v) starch and 2% (w/v) agar. Above this an agar cap consisting of 50 mL of 5% (w/v) agar and a top layer of liquid media (glucose free MSM). c) Diffusion of starch from the starch-agar layer into the liquid media from flasks containing no cells measured by starch degradation assay of liquid media samples over time with concentration calculated using a standard curve.
